# Chemical Fingerprint of Free Polyphenols and Antioxidant Activity in Dietary Fruits and Vegetables Using a Non-Targeted Approach Based on QuEChERS Ultrasound-Assisted Extraction Combined with UHPLC-PDA

**DOI:** 10.3390/antiox9040305

**Published:** 2020-04-09

**Authors:** Joselin Aguiar, João L. Gonçalves, Vera L. Alves, José S. Câmara

**Affiliations:** 1 CQM—Centro de Química da Madeira, Universidade da Madeira, Campus Universitário da Penteada, 9020-105 Funchal, Portugal; joselin.aguiar@staff.uma.pt (J.A.); jluis@staff.uma.pt (J.L.G.); vera.alves@staff.uma.pt (V.L.A.); 2 Faculdade de Ciências Exactas e da Engenharia, Universidade da Madeira, Campus da Penteada, 9020-105 Funchal, Portugal

**Keywords:** bioactive compounds, polyphenols, antioxidants, fruits, vegetables, QuEChERS-USAE

## Abstract

Fruits and vegetables are considered a good source of antioxidants, which are beneficial in protecting the human body against damage induced by free radicals and other reactive oxygen (ROS) and nitrogen (RNS) species. In this work, we aimed to evaluate the integral antioxidant activity (AOA) and determine individual polyphenols in fruits and vegetables of frequent consumption. For this purpose, an innovative and high throughput analytical approach based on original QuEChERS assisted by ultrasound extraction (USAE), instead of the manual agitation used in the classical procedure, was optimized and implemented for the isolation of polyphenols. The total phenolic content (TPC), flavonoids, anthocyanins, and betalains were evaluated using different spectrophotometric assays. In addition, free radical scavenging by methods 2,2’-azinobis-(3-ethylbenzothiazoline-6-sulfonate) (ABTS) and 2,2′-diphenyl-1-picrylhydrazyl (DPPH) and ferric reducing antioxidant power (FRAP) were used to estimate the AOA of the investigated fruit and vegetable extracts. Red onion, tamarillo, and beetroot were the samples with the highest AOA. The quantification and identification of free low molecular weight polyphenols from QuEChERS-USAE extracts was carried out by ultra-high-pressure liquid chromatography equipped with a photodiode array detection system (UHPLC-PDA). Catechin was the most abundant polyphenol, followed by gentisic and ferulic acids, mainly in the watercress sample. In relation to flavonols, quercetin and kaempferol were found mostly in onion samples, and in small quantities in tomato and watercress. The improved analytical approach, QuEChERS-USAE/UHPLC-PDA, offers an attractive alternative for the analysis of polyphenols from fruit and vegetable samples, providing several advantages over traditional extraction techniques, in terms of reproducibility, simplicity, low cost, analysis speed, and analytical performance.

## 1. Introduction

The significant increase in the duration of the biologically active life associated with an unhealthy lifestyle, expressed in the harmful use of alcohol, tobacco use, physical inactivity, and an unhealthy diet, constitutes the most important risk factor for noncommunicable diseases (NCDs), including cardiovascular diseases (CVDs), cancer, respiratory diseases, and diabetes. It is estimated that 71 million people die each year from NCDs, and these four groups of diseases are responsible for about 80% of all premature NCD deaths. Increasing evidence suggests that a well-balanced diet, rich in fruits and vegetables, can prevent a large number of diseases, such a, cancer [1], CVD [2,3], poor cognitive performance [4,5], and other diet-related diseases [6,7]. In this context, the World Health Organization (WHO) recommends a daily intake of five to eight portions (400–600 g) of fruits and vegetables to reduce the risk of occurrence of diet-related diseases, since they constitute one of the main sources of vitamins, minerals, fiber, and a large number of phytochemicals responsible for organoleptic and biological properties [8].

Among the thousands of phytochemicals found in our diets, polyphenols stand out as the most important group of natural antioxidants. They exhibit a wide range of beneficial effects, including hypolipidemic [9], antioxidative [10], anti-carcinogenic [11], antiproliferative [12], and anti-inflammatory effects [13,14]. As antioxidants, polyphenols may protect cell constituents against oxidative damage. Their capacity to scavenge free radicals, which are constantly generated from cell metabolism and can cause damage to biologically relevant molecules, such as DNA, proteins, carbohydrates, and lipids, helps to maintain the homeostatic balance for proper functioning of the body [15].

Currently, many experimental studies and clinical evidence suggest that polyphenols have a positive impact on the vascular system by inhibiting the oxidation of low-density lipoproteins (LDLs) [16], reducing the formation of atherosclerotic lesions [17], inhibiting platelet aggregation [18], decreasing the expression of vascular cell adhesion molecules [19], improving endothelial function [20], and reducing blood pressure [21]. Moreover, polyphenols exert a variety of anti-carcinogenic effects, including their inhibitory effects on cancer cell proliferation [22,23], inhibition of tumor cell invasion and angiogenesis [24], and ability to induce apoptosis in tumor cells [25].

Apart from their biological properties, polyphenols are also of great interest in the pharmaceutical, cosmetic, and, especially, in food industries, since they can also be used as substitutes for synthetic antioxidants, providing protection against oxidative degradation [26,27].

Given the evidence on the health benefits of polyphenols and their influence on food quality, the monitoring of polyphenols in fruits and vegetables is a priority task of analytical and food chemistry. Several methods and approaches have been proposed for the study of antioxidant activity (AOA) and the quantitative determination of phytochemicals. However, due to the great variety of compounds that can be found in food samples, with different sizes, polarity, and forms (glycosylated or in their aglycone form), the analysis of polyphenols is a relatively complex task [28]. On the other hand, many polyphenols may occur at low concentration levels, which makes their determination quite challenging. Recently, new extraction and clean-up approaches have been developed in order to simplify sample preparation, moving towards more environmentally friendly techniques and more efficient analysis [28]. One of the significant advances in sample preparation was the introduction of the ‘Quick, Easy, Cheap, Effective, Rugged and Safe’ (QuEChERS) methodology, which combines an initial salting out liquid–liquid extraction (SALLE) with a dispersive solid-phase extraction (*d*-SPE) clean-up [29]. This methodology was originally developed to extract pesticides from fruits and vegetables. However, due to its high flexibility, nowadays, the QuEChERS concept is used in the analysis of many different types of organic compounds in almost all types of agri-food, environmental, and biological samples [30].

Recently, the QuEChERS methodology was successfully applied to the extraction of polyphenols from vegetable samples [31,32] and baby foods [28]. In these methods, the extraction of polyphenols requires sample homogenization, which is particularly difficult when the sample is in a solid form. For the treatment of solid samples, a good alternative is the use of ultrasound agitation, as it forms cavitation of the small bubbles in the solvent due to the passage of ultrasound waves, allowing greater penetration of the solvent into the sample, and consequently, increases the surface area [33,34]. On the other hand, the cavitation phenomenon is capable of damaging the cell walls of the plant matrix, favoring the release of bioactive compounds and providing better extraction efficiencies [35].

Therefore, the main goal of this study was to optimize and validate a novel and improved methodology based on QuEChERS ultrasound-assisted extraction (USAE) and ultra-high-pressure liquid chromatography equipped with a photodiode array detection system (UHPLC-PDA) for the extraction and quantification of polyphenols from fruits and vegetables. Some important parameters that might affect the extraction efficiency, namely the sonication time and partitioning solvents, were investigated and optimized. In addition, the total phenolic content (TPC), total flavonoid content (TFC), total anthocyanin content (TAC), and total betalain content (TBC) of each fruit and vegetable were assayed through spectrophotometric methods, as well as the AOA using different assays, including those based on free radical-scavenging activity assays (2,2-diphenyl-1-picrylhydrazyl (DPPH) and 2,2′-azino-bis-3-ethylbenzthiazoline-6-sulfonic acid (ABTS)) and reducing power (ferric reducing antioxidant power, FRAP).

## 2. Materials and Methods

### 2.1. Reagents, Materials, and Standards

All chemicals and reagents were of analytical grade. Methanol HPLC-grade (MeOH), acetonitrile HPLC-grade (ACN), ethyl acetate (EtAc), and formic acid (FA) were obtained from Fischer Scientific (Loughborough, UK). 2,2’-Azinobis-(3-ethylbenzothiazoline-6-sulfonate) (ABTS), aluminum chloride (AlCl_3_), and sodium phosphate dibasic dodecahydrate (Na_2_HPO_4_·12H_2_O) were purchased from Riedel-de Haën (Denmark), whereas 2,2′-diphenyl-1-picrylhydrazyl (DPPH), 2,4,6-tri-(2-pyridyl)-s-triazine (TPTZ), 2-thiobarbituric acid, trichloroacetic acid, ferric chloride (FeCl_3_), sodium chloride (NaCl), trisodium citrate dihydrate (Na_3_C_6_H_5_O_7_·2H_2_O), sodium citrate dibasic sesquihydrate (Na_2_C_6_H_6_O_7_·1.5H_2_O), and magnesium sulfate (MgSO_4_) were purchased from Sigma-Aldrich (Germany). Anhydrous sodium carbonate (Na_2_CO_3_), sodium acetate trihydrate (C_2_H_3_NaO_2_·3H_2_O), sodium hydroxide (NaOH ), and potassium dihydrogen phosphate (KH_2_PO_4_) were obtained from Panreac Quimica S.A (Spain). Folin-Ciocalteu reagent was purchased from Fluka (Switzerland), whereas extra pure sodium nitrate (NaNO_3_) and glacial acetic acid (CH_3_COOH) were obtained from Merck (Germany).

For the identification and quantification of polyphenols, the following analytical standards were used: Gallic acid (98.0%), gentisic acid (≥98.0%), *m*-coumaric acid (≥98.0%), o-coumaric acid (≥97.0%), p-coumaric acid (99%), cinnamic acid (≥99.0%), ferulic acid (≥99.0%), and vanillic acid (97%) were purchased from Fluka (Germany). Kaempferol (≥97%), (+)-catechin (≥99.0%), and protocatechuic acid (98%) were purchased from Sigma-Aldrich (Germany). Quercetin dihydrate (99.0%) was obtained from Riedel-de Haën (Denmark) and syringaldehyde (98%) from Acros Organics (Belgium). Internal standard, 6-hydroxy-2,5,7,8-tetramethylchroman-2-carboxylic acid (Trolox), was acquired from Fluka Biochemica AG (Buchs, Switzerland).

Ultrapure water (18 MΩ cm) was obtained by means of a Milli-Q water purification system (Millipore, Milford, MA, USA) and was used for preparing the mobile phase and other aqueous solutions. All samples and standards were filtered through 0.22-µm polytetrafluoroethylene (PTFE) membrane filters. Sorbents (50-µm particle size) for d-SPE, including trifunctionally bonded C_18_ silica, primary-secondary amine (PSA), anhydrous MgSO_4_, and the QuEChERS extraction/partitioning tubes containing the buffer salts and the clean-up tubes were acquired from Waters (Milford, MA, USA).

### 2.2. Preparation of Standard Solutions

Individual stock solutions of each polyphenol were prepared in MeOH at a concentration of 1000 µg/mL, and aliquoted in 2-mL vials, and stored at −20 °C, in the dark. Multicomponent standard solution was prepared to a final concentration of 25 µg/mL by dilution of the initial methanolic solutions and were used to optimize the extraction conditions. For validation purposes, working standard solutions at different concentration levels (5–25 µg/mL) were prepared daily by diluting the stock solutions with MeOH. The target analytes were chosen according to their importance and/or relevance to fruit and vegetable quality.

### 2.3. Fruit and Vegetable Samples

Several fruit and vegetable samples, namely tomato (*Solanum lycopersicum*), tamarillo (*Solanum betaceum*), watercress (*Rorippa nasturtium-aquaticum*), broccoli (*Brassica oleracea*), spinach (*Spinacia oleracea*), white and orange carrot (*Daucus carota*), beetroot (*Beta vulgaris*), yellow and red onion (*Allium cepa*), and garlic (*Allium sativum*), were purchased from a local market in Funchal, Madeira Island, Portugal. For each sample, 1 kg was randomly sampled from the market shelves, discarding vegetables with unpleasant appearance. Each sample was washed in tape water and all inedible parts were removed. Then, 500 g of each sample were placed in a commercial immersion blender (200 W, Krups) to obtain the respective pulps and stored at −20 °C in the dark until analysis.

### 2.4. QuEChERS Assisted by Ultrasound for the Extraction of Polyphenols

The procedure used to isolate the polyphenols from target fruits and vegetables was based on the original QuEChERS procedure adapted from the methodology reported by Anastassiades and Lehotay [29] to which an innovative step based on the extraction assisted by ultrasound, instead of the manual agitation used in the original procedure, was included. This step was included to improve the extraction efficiency of the procedure and increase the reproducibility between extractions. Several extraction-influencing QuEChERS-USAE parameters were evaluated and optimized and the method was validated according to the International Union of Pure and Applied Chemistry (IUPAC) guidelines using the optimized parameters.

#### 2.4.1. Selection of Sonication Time and Extraction Solvent

The QuEChERS-USAE analytical approach is an improved strategy adapted from the methodology reported by Anastassiades and Lehotay [29]. In order to obtain the highest extraction efficiency of polyphenols, the influence of the sonication time and partitioning solvent were evaluated. For the sonication time, 1, 5, and 10 min were evaluated and compared with the manual agitation (2 min), the time used for the original QuEChERS. Once the best extraction time was selected, the partitioning solvents ACN, EtAc, MeOH, ACN:EtAC (50:50, *v/v*), MeOH:ACN (50:50, *v/v*), MeOH:EtAc (50:50, *v/v*), and MeOH:H_2_O (80:20, *v/v*) were tested. Orange carrot (*Daucus carota* L.) samples were selected as the matrix for the optimization of the QuEChERS-USAE procedure.

#### 2.4.2. QuEChERS-USAE and Clean-Up Procedure

**Figure 1:**
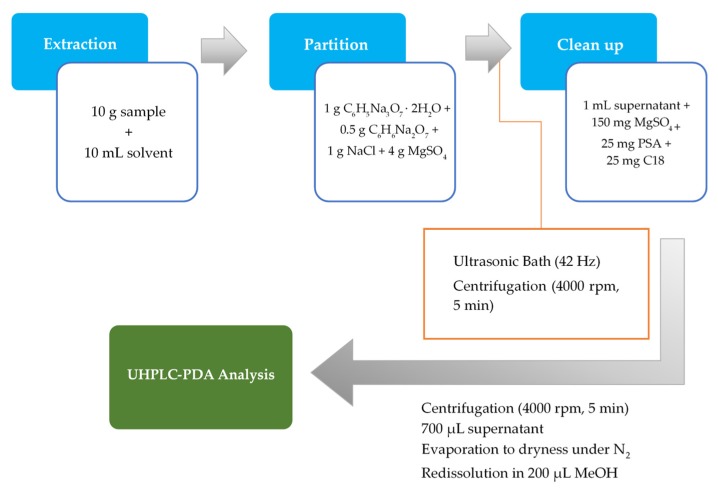
Schematic representation of the novel approach based on the QuEChERS-USAE procedure.

In the first step, 10 g of a thoroughly homogenized solid sample of fruits and vegetables were weighted in a 50-mL PTFE centrifuge tube. Then, 10 mL of MeOH were added and the tube was shaken vigorously for 2 min, ensuring a total interaction between the solvent and sample. Buffered salts, disodium hydrogencitrate sesquihydrate (0.5 g), trisodium citrate dihydrate (1 g), sodium chloride (1 g), and MgSO_4_ (4 g) were added into the homogenized mixture and placed in an ultrasonic bath for 5 min. Afterward, the mixture was centrifuged at 4000 rpm for 5 min, and 1 mL of supernatant was transferred to a 2-mL PTFE *d*-SPE clean-up tube containing PSA (25 mg), C_18_ sorbent (25 mg), and MgSO_4_ (150 mg). The mixture was then vortexed for 30 s and centrifuged at 4000 rpm for 5 min and the supernatant (700 µL) was filtered through a 0.22-µm PTFE filter and dried under a gentle nitrogen stream. Finally, the residue was dissolved in 200 µL of MeOH for subsequent analysis on the UHPLC-PDA system. The extraction process is described schematically in Figure 1.

### 2.5. Evaluation of the Bioactive Potential of QuEChERS-USAE Extracts.

#### 2.5.1. Total Phenolic Content (TPC)

The TPC of fruit and vegetable QuEChERS-USAE extracts was determined by the Folin Ciocalteu’s colorimetric method described by Singleton et al. [36] with some modifications. Briefly, 50 µL of the sample extracts were mixed with 3 mL of distilled water and 250 µL of Folin Ciocalteau reagent. After 5 min, 750 μL of 20% (*w/v*) Na_2_CO_3_ were added and the resulting mixture was vortexed for 2 min and incubated at room temperature in the dark for 30 min. The absorbance of the solution was measured at 750 nm using a UV-Vis Spectrophotometer (Perkin Elmer Lambda 25, ILC-Instrumentos de Laboratório e Científicos, Lda., Portugal). Gallic acid (25–400 mg/L) was used as standard to prepare a calibration curve, from which TPC was determined in terms of mg of gallic acid equivalent in one gram of fresh mass (mg GAE/g).

#### 2.5.2. Total Flavonoid Content (TFC)

The TFC of fruit and vegetable QuEChERS-USAE extracts was determined using the aluminum chloride colorimetric assay described by Marinova and Ribarova [37], with some modifications. Briefly, an aliquot of 1 mL of sample was added to a 10-mL volumetric flask containing 4 mL of distilled water and 300 μL of 5% NaNO_2_. The mixture was allowed to stand for 5 min, and then 300 µL of 10% AlCl_3_ were added. After 1 min, 2 mL of NaOH (1M) were added to the mixture and the total volume was increased to 10 mL with distilled water. The resulting mixture was shaken vigorously for 2 min and the absorbance was measured at 415 nm. It is important to note that all determinations were carried out in triplicate. The calibration curve was constructed using quercetin standard solution at different concentration levels (10 to 400 mg/L), and the TFC of fruits and vegetables was expressed as mg of quercetin equivalent per gram of fresh mass (mg QE/g).

#### 2.5.3. Total Anthocyanin Content (TAC)

The TAC of fruit and vegetable extracts was determined by the pH differential method proposed by Giusti and Wrolstad [38]. Each sample extract was diluted separately at a 1:10 ratio with 0.025 M potassium chloride buffer (pH = 1) and 0.4 M sodium acetate buffer (pH = 4.5). After an equilibration period of 15 min at room temperature, the absorbance of each solution was measured at 520 and 700 nm. The TAC was expressed as cyanidin-3-glucoside equivalents per gram of fresh mass (mg C3GE/g) using the following equation:

TAC (mg C3GE/g) = (A × MW × DF × V × 1000)/(ε × *l* × *m*), (1)

where A is the absorbance calculated as A = (A_520_ – A_700_)_pH 1_ – (A_520_ – A_700_)_pH 4.5_, MW is the molecular weight of cyanidin-3-glucoside (449.2), DF is the dilution factor, V is the volume of extract (L), 1000 is the conversion factor from gram to milligram, *ε* is the molar absorptivity (26,900 L/mol.cm), *l* is the cell path length (1 cm), and *m* is the sample mass (g).

#### 2.5.4. Total Betalain Content (TBC)

For the beetroot sample, the TBC were determined according to the procedure described by Koubaier et al. [39] with slight modifications. For this purpose, beetroot extract was diluted to a proper concentration in 0.05 M phosphate buffer (pH 6.5) and the absorbance was measured at 480 and 538 nm for betanin and vulgaxanthin I, respectively. The betalain content (BC) was calculated using the following formula:

BC (mg/g) = (A × MW × DF × V × 1000)/(ε × *l* × m), (2)

were A is the absorption at 538 and 480 nm for betacyanins and betaxanthins, respectively; MW is the molecular weight (339 g/mol for vulgaxanthin and 550 g/mol for betanin); DF is the dilution factor; V is the volume of extract (L); ε is the molar extinction coefficients (48,000 L/mol.cm at λ = 480 nm for vulgaxanthin and 60,000 L/mol.cm at λ = 538 nm for betanin); *l* is the cell path length (1 cm); and *m* is the sample mass (g).

### 2.6. Evaluation of the Antioxidant Abilities of the Investigated Extracts

#### 2.6.1. DPPH Assays

The DPPH radical scavenging activity of fruit and vegetable extracts was determined according to the method described by Thaipong et al. [40], with some modifications. Briefly, 150 µL of extract (QuEChERS-USAE) were mixed with 2850 μL of methanolic solution of DPPH (60 µM). The mixture was shaken vigorously and allowed to stand for 30 min in the dark at room temperature. Afterward, the absorbance values of these solutions were recorded spectrophotometrically at 515 nm using a control containing the same concentration of DPPH radicals. The radical scavenging activity (RSA) was determined as a percentage of the DPPH· discoloration using the following formula:

RSA (%) = [(A_control_ − A_sample_)/A_control_] × 100, (3)

where A_control_ is the absorbance of the DPPH radical in methanol and A_sample_ is the absorbance of the DPPH radical solution mixed with the sample extract. A calibration curve was obtained using Trolox standard solution at different concentrations (10–1200 μM). The results obtained were also expressed as µM Trolox equivalent per gram of fresh mass (µM TE/g). All samples were carried out in triplicate.

#### 2.6.2. ABTS Assay

The ABTS radical scavenging activity of the QuEChERS-USAE extracts was determined according to the method described by Thaipong et al. [40], with some modifications. The ABTS radical cation (ABTS•^+^) was prepared by mixing a 7.4 mM ABTS solution with a 2.6 mM potassium persulfate solution at a ratio of 1:1 (*v/v*) and stored at room temperature in the dark for 12 h. ABTS•^+^ stock solution was diluted before use to an absorbance of 0.700 ± 0.020 at 734 nm with MeOH. Then, 2850 μL of the ABTS•^+^ solution were mixed with 150 µL of fruit and vegetable extracts and allowed to react for 30 min in the dark at room temperature. Finally, the absorbance was taken at 734 nm. The ABTS•^+^ scavenging capacity of the extract was determined using the following formula:

ABTS•^+^ scavenging effect (%) = [(A_control_ − A_sample_)/A_control_] × 100, (4)

where A_control_ is the absorbance of the ABTS•^+^ solution in methanol and A_sample_ is the absorbance of the ABTS•^+^ solution mixed with the sample extract. The calibration curve between the % ABTS•^+^ scavenging capacity and known solutions of Trolox (10–400 μM) was then established and the results were expressed as µM Trolox equivalent per gram of fresh mass (µM TE/g).

#### 2.6.3. FRAP Assay

The ferric reducing antioxidant power (FRAP) of each fruit and vegetable extract (QuEChERS-USAE) was determined according to the method described by Thaipong et al. [40]. Succinctly, 150 µL of sample were added to 2850 µL of FRAP solution, which was previously prepared by mixing 25 mL of acetate buffer (300 mM) at pH 3.6, 2.5 mL of 10 mM TPTZ solution prepared in HCl (40 mM), and 2.5 mL of FeCl_3_·6H_2_O (20 mM) and then warmed at 37 °C. The resulting mixture was reacted for 30 min at 37 °C in the darkness and then the absorbance of the colored product (ferrous tripyridyltriazine complex) was recorded at 593 nm. A calibration curve was prepared with Trolox in a concentration ranging from 10 to 400 μM, and the results were expressed as µM Trolox equivalent per gram of fresh mass (µM TE/g).

### 2.7. UHPLC-PDA Analysis and Operating Conditions

The identification and quantification of phenolic compounds was performed on a Waters ultra-high-pressure liquid chromatography Acquity system (UPLC, Acquity H-Class) (Milford, MA, USA) equipped with a 2996 PDA detector. Separation was achieved with an ACQUITY HSS T3 analytical column (100 mm × 2.1 mm, 1.8 μm particle size) protected with an Acquity UPLC^TM^ HSS T3 Van Guard^TM^ Pre-column (Waters, Milford, MA, USA). The column oven temperature was kept at 40 °C. A binary mobile phase combining 0.1% aqueous formic acid solution (solvent A) and acetonitrile (solvent B) was used with a gradient program, as follows: 80% A (0 min), 60% A (3 min), 55% A (6 min), 30% A (8–10 min), and 80% A (12–14 min), followed by a re-equilibration time of 2 min prior to the next injection. The flow rate was 250 μL/min and the injection volume of both standards and samples was 2 μL. The UV detection wavelength was set to the maximum of absorbance for the compounds of interest and the Empower 2 software (Milford, MA, USA) was used for chromatographic data gathering and integration of chromatograms. The identification of polyphenols was based on the retention time and UV spectrum.

### 2.8. Analytical Method Validation.

Validation of the QuEChERS-USAE/UHPLC-PDA methodology for the quantification of polyphenols in fruits and vegetables involved the assessment of the selectivity, linearity, limits of detection and quantification (LOD and LOQ), intra-day and inter-day precision, trueness, and extraction efficiency. The selectivity of the method was assessed by the analysis of fruit and vegetable samples by the QuEChERS-USAE/UHPLC-PDA methodology and compared with a 10 μg/mL standard solution. The absence of interfering peaks at the elution times of the target analytes proves that the method is selective.

Linearity was assessed by constructing a calibration curve for each analyte with five calibration points (*n* = 5) with concentrations ranging from 5 to 25 μg/mL. This working range was selected taking into account the sensitivity of the UHPLC detector and the range of polyphenol concentrations commonly found in fruits and vegetables. For each calibration solution, the internal standard Trolox (10 μg/mL) was added and the calibration curves were obtained by plotting the peak–area ratio between each analyte and the internal standard (area_analyte_/area_IS_) versus the corresponding analyte concentration. Mandel’s fitting test was considered to complement the linearity of the method.

The limits of detection (LOD) and the limits of quantification (LOQ) were assessed on the basis of the concentration that produced a signal-to-noise (S/N) ratio equal or higher than 3 and 10, respectively.

Regarding the precision of the method, it was determined in terms of the intra-day and inter-day precision in three different concentration levels (5, 15, and 25 μg/mL). Intra-day precision was assessed by the application of the developed methodology on the same day, by the same analyst (experimental replicates *n* = 6), while inter-day precision was evaluated with a similar procedure on different days (*n* = 18). The results were expressed in terms of the relative standard deviation (% RSD). Concerning the trueness of the method, it was estimated in terms of the relative bias, which is determined as the difference between the expectation of the test results and an accepted reference value. In practice, the trueness validation procedure was carried out by spiking the sample at three concentration levels: Low (5 μg/mL), medium (15 μg/mL), and high (25 μg/mL) levels. Samples were analyzed in triplicate over a period of three days and the trueness of the method was acceded using the relative bias formula:

Relative bias (%) = [(x − x_ref_)/x_ref_] × 100, (5)

where x denotes the mean of the results and x_ref_ the reference value.

The extraction efficiency was expressed as the recovery percentage (%) and was assessed by spiking the sample in triplicate at three concentration levels (low, medium, and high) and subjecting them to the QuEChERS procedure. The recovery values were determined by a comparison of the areas of the spiked sample with the areas of the simulated sample (sample spiked at the same concentration levels but at the end of the extraction process prior to UHPLC-PDA analysis).

## 3. Results and Discussion

### 3.1. Optimization of QuEChERS-USAE: Selection of the Sonication Time and Extraction Solvent

**Figure 2:**
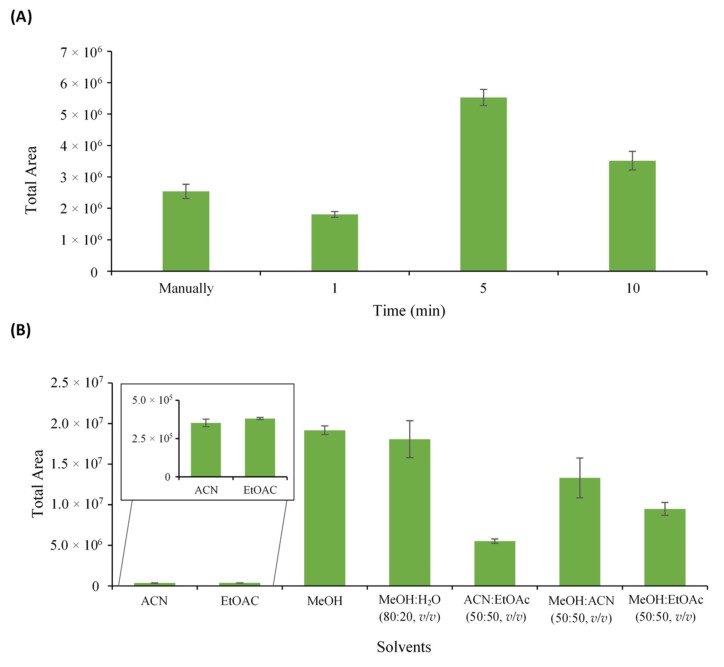
Optimization of different parameters that influence the extraction efficiency. (**A**) ultrasound time and (**B**) extraction solvents. Error bars represent the standard deviation of sample replicates (*n* = 3).

For the optimization of the QuEChERS procedure, orange carrot was selected as the matrix, as described in Section 2.4. In order to obtain the highest extraction efficiency of polyphenols, different ultrasound extraction times were evaluated, namely 1, 5, and 10 min, and compared with manual agitation. Figure 2A shows the obtained results.

According to the results, USAE presented a better extraction efficiency compared to manual agitation. This was expected since the application of the ultrasonic waves produced during the process generates cavitation and consequent rupture of the cell walls, leading to the extraction of phenolic compounds from the sample to the solvent [41,42].

Regarding the effect of the different USAE times, the results revealed that between 1 and 5 min, there was an increase of the total area of around 67%, while between 5 and 10 min, there was a decrease of approximately 36% of the total area. These results indicate that the high ultrasound frequency facilitates the diffusion of phenolic compounds from the plant material to the solvent in a short time. However, if exposure to the ultrasonic waves is prolonged, the degradation of antioxidant compounds may occur [43,44]. Thus, 5 min was used in the subsequent experiments.

In addition, to improve the extraction efficiency of polyphenols in the samples, different partitioning solvents, namely MeOH, ACN, EtOAC, and different combinations of them, were tested and compared. From the comparison of the graph presented in Figure 2B, it was found that 100% MeOH provided the best results in terms of repeatability and the chromatographic response (total area). On the other hand, 100% ACN and 100% EtAc showed the lowest extraction efficiency for the target compounds. Thus, MeOH was the solvent selected to extract the polyphenols from fruits and vegetables.

### 3.2. Determination of the Phytochemical Composition

Among the wide range of phytochemical compounds that constitute fruits and vegetables, phenolic compounds, flavonoids, anthocyanins, and betalains play a prominent role due to their antioxidant and free radical scavenging activities. Table 1 shows the total content of phenolic compounds, flavonoids, anthocyanins, and betalains present in the analyzed samples.

The TPC found in all samples ranged from 0.39 mg GAE/g fresh mass for orange carrot to 1.33 mg GAE/g fresh mass for red onion. Comparing the two onion varieties, the red onion showed the highest content, which is in agreement with the results obtained by Nile and Park [45]. In this study, the methanolic extracts from the bulbs of three onion varieties (red, white, and yellow) were analyzed, with red onion being the variety with the highest TPC, followed by the extract of yellow onion and finally the white variety. According to Shahidi et al. [46], this difference may be associated with the high content of anthocyanins present in red onion, thus contributing to the higher TPC in this variety.

In Table 1, it was also possible to verify that the methanolic extracts of watercress, spinach, and broccoli had the highest levels of flavonoids. In general, dark green leafy vegetables, such as broccoli, watercress, and spinach, as well as yellow or red fruits and vegetables, are very rich in flavonoids [47,48]. When comparing the obtained results with those reported in the literature, it was observed that *Brassica* plants, such as broccoli and watercress, presented much lower flavonoid contents than those obtained by Agarwal et al. [49] (broccoli, 13.98 mg QE/g vegetable) and Aires et al. [50] (watercress, 5.6 mg CatE/g dried weight). This difference may be associated with some factors, such as geographic variation, harvest time, and environmental and agronomic conditions [51,52].

Regarding the TAC, it was found that the methanolic extracts of tamarillo, watercress, and red onion had the highest anthocyanin content, with tamarillo being the sample with the higher value (17.3 μg C3GE/g fresh mass). Tamarillo is a tropical fruit characterized by its oval shape, and its color varies from yellow to red. This fruit is considered to be a very rich source of natural pigments, such as anthocyanins, which contribute to its antioxidant potential [53]. For the red onion sample, several studies have revealed that flavonols and anthocyanins are the main subclasses of flavonoids in this vegetable [54]. In this study, the anthocyanin content value for the red onion sample was 5.13 μg C3GE/g fresh mass, which is in agreement with the results obtained by Rodrigues et al. [54], where the content of these compounds ranged from 0.5 to 5.9 μg/g fresh mass.

For the beetroot extract, no anthocyanins were found in this sample. In fact, the intense red-purple color of beetroots comes from a particular family of pigments called betalains. Betalains are water-soluble nitrogen-containing pigments, which are synthesized from the amino acid tyrosine into two structural groups: The red-violet betacyanins and the yellow-orange betaxanthins. In the present study, the TBC for the beetroot extract was 899.1 ± 10.9 μg betalains/g fresh mass. The amount of betacyanins found in this sample was 68.2% of the TBC, which corresponds to twice the amount of betaxanthins (31.8%). Lee et al. [55] also found a similar TBC in their studies, where the beetroots produced in the field had TBC values ranging from 650 to 800 μg betalain/g fresh mass.

### 3.3. In Vitro Evaluation of Antioxidant Abilities of the Investigated Extracts

**Figure 3:**
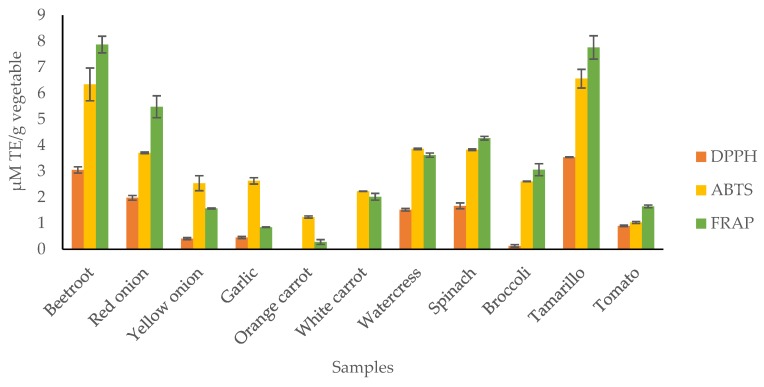
Antioxidant abilities of the investigated fruit and vegetable QuEChERS-USAE extracts determined by free radical scavenging assays (DPPH and ABTS) and reducing power (FRAP) spectrophotomeric assays.

Given the complexity of bioactive compounds in fruits and vegetables, it is always advisable to use more than one method to evaluate the antioxidant capacity of these matrices. Figure 3 presents the results obtained in all AOA assays performed.

According to the results, it was possible to verify that all extracts showed antioxidant activity, mainly the beetroot, tamarillo, and red onion extracts, which were more efficient in neutralizing DPPH• and ABTS•^+^ radicals and in their ferric reducing ability. These results were expected due to the concentrations of bioactive compounds found in these matrices. Tamarillo and red onion are plant species rich in phenolic compounds, such as anthocyanins, which have been strongly related to the antioxidant activity of these species [56,57]. Beetroot, in turn, is a vegetable characterized by the presence of betalains, which have high antioxidant properties [58,59]. Although individual bioactive compounds may provide the antioxidant capacity, when considered together, the effect of the interaction can be enhanced, producing additive and/or synergistic effects, which have been proposed to be responsible for the potent antioxidant and anticancer activities of the phytochemicals in fruits and vegetables [60,61].

### 3.4. QuEChERS-USAE for the Extraction of Polyphenols

#### 3.4.1. Method Validation

To demonstrate the feasibility and applicability of the QuEChERS-USAE/UHPLC-PDA procedure for the quantification of polyphenols in fruits and vegetables, the performance of the procedure was evaluated through its validation following the IUPAC guidelines. The method selectivity, linearity, LOD, LOQ, intra-day and inter-day precision, trueness, and extraction efficiency were determined. Regarding the method selectivity, no interfering peaks were detected at the retention time of each analyte at their maximum absorbance wavelengths (Table 2).

For the linearity evaluation, the calibration curves for each analyte were determined by plotting the ratio between the analyte and internal standard peak areas against the analyte concentration (area_analyte_/area_IS_ vs. concentration). The least-squares regression analysis provided good determination coefficients (*R*^2^ ≥ 0.99, Table 2) in the concentration range studied. Moreover, it was possible to demonstrate through the Mandel’s fitting test that the linear model provided a significantly better fit (TV ≤ F (0.95; 1; N-3)) than the quadratic model.

Regarding the LOD and LOQ, both limits were determined from theoretical calculations of the lowest concentration level, obtaining an S/N ratio equal or higher than 3 and 10, respectively. The QuEChERS/UHLPC-PDA methodology showed, in general, low LODs (Table 3), ranging from 0.04 µg/mL for gentisic acid to 0.20 µg/mL for *p*-coumaric acid, while LOQs ranged between 0.12 and 0.61 µg/mL for both compounds, respectively.

The precision and trueness were evaluated at three concentration levels and the results are shown in Table 2. The trueness values were expressed in terms of the relative bias (%) and as can be seen in Table 3, the relative bias of the developed method was found to be acceptable since it was relatively close to 0, except at the lowest concentration levels for which it was around approximately 20%. Precision was measured through intra-day and inter-day studies and results were represented by the RSD at each fortification level for each compound. The obtained RSD values were acceptable for all the analytes, as they were all lower than 20% (between 2% and 20%).

The recovery study was carried out by spiking the sample, in triplicate, at the same three concentration levels analyzed for the precision and trueness. The results revealed that the recoveries ranged from 59% to 97% for the lowest concentration, while for the medium and high concentrations, the values ranged from 68–102% to 86–112%, respectively. Often, recovery rates above 70% are acceptable. However, when the recovery rate is low but the value is consistent (showing good precision), a recovery average below 70% may be acceptable. Dadgar and Burnett [62] state that sometimes it is preferable to sacrifice the method’s recovery rate for greater selectivity as long as the methodology demonstrates adequate sensitivity, precision, and accuracy. In this context, it can be concluded that the recovery results are satisfactory, with QuEChERS-USAE being based on an analytical technique suitable for the extraction of phenolic compounds from fruits and vegetables.

#### 3.4.2. Application of QuEChERS-USAE/UHPLC-PDA for the Analysis of Polyphenols in Fruit and Vegetable Samples

**Figure 4:**
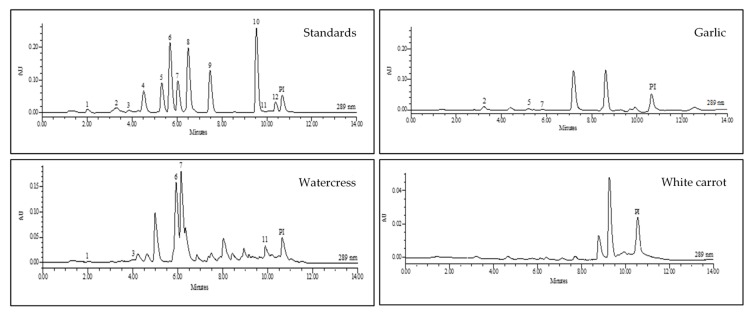
Representative typical QuEChERS-USAE/UHPLC-PDA chromatograms for polyphenol standards and for some studied vegetables. For the peak assignment, see Table 2.

After analytical validation, the optimized method was applied to 11 vegetable and fruit samples, namely beetroot, watercress, spinach, broccoli, garlic, tomato, tamarillo, white and orange carrot, and two varieties of onion (yellow and red). The chromatograms obtained for all samples can be seen in Figure 4 and the mean concentrations of phenolic compounds through USAE based on the QuEChERS/UHPLC-PDA methodology are described in Table 3.

In general, the obtained results are satisfactory for almost all the analyzed vegetables, except for white carrot, in which polyphenols could not be detected. The absence of these compounds in the matrix may be associated with several factors, including the variety of the vegetable not containing the studied polyphenols or the method not being sensitive enough to detect the trace amounts of these compounds in this matrix. When comparing these results with other studies [63], it was found that none of the studied polyphenols were reported in the literature for white carrots.

Among all the polyphenols analyzed, catechin was the most abundant polyphenol since it was detected in five samples (garlic, orange carrot, spinach, broccoli, tamarillo, and tomato), followed by gentisic and ferulic acids. Catechin, a flavan-3-ol belonging to the flavonoid group, is widely distributed in a wide variety of foods, including fruits and vegetables, and has been identified and quantified in garlic, carrot, spinach, broccoli, and mainly in tomato and tamarillo samples.

Regarding the composition of phenolic acids present in the matrices analyzed, gentisic acid, ferulic acid, and protocatechuic acid were undoubtedly the most outstanding. Gentisic acid, such as protocatechuic acid, is derived from hydroxybenzoic acids that can be found in certain types of foods. Generally, the content of these compounds in edible plants is very low, except for some types of fruits and some varieties of vegetables, which may have concentrations of several tens of milligrams per kilogram fresh weight [64].

Hydroxycinnamic acids, especially *p*-coumaric, caffeic, ferulic, and synaptic acids, are more abundant than hydroxybenzoic acids. In the present work, hydroxycinnamic and hydroxybenzoic acids were identified in almost all the samples studied except for broccoli, white carrot, and both onion varieties. Regarding the diversity of these compounds in the matrices analyzed, orange carrot was the vegetable with the highest number of phenolic acids identified.

Regarding the group of flavonols studied, quercetin and kaempferol were found mainly in the onion samples. This result was expected as both compounds and their glycosidic derivatives are often found in these vegetables [65,66].

The biological activity of these polyphenols found in the studied fruits and vegetables have often been evaluated in vitro on pure enzymes, cultured cells, or isolated tissues [67]. The main reason for this interest is the recognition of the antioxidant properties of polyphenols, which have been demonstrated in the prevention of several diseases associated with oxidative stress, such as cancer and cardiovascular and neurodegenerative diseases. Catechin, for example, was identified in six of our samples and presents several biological properties, such as anti-inflammatory, antioxidant, and antibacterial properties [68]. Table 4 shows the polyphenols identified in our vegetable samples and their respective biological properties.

## 4. Conclusions

In this work, the bioactive composition of 11 fruits and vegetables and their antioxidant capacity was evaluated. The results of this study revealed that the QuEChERS-USAE extracts of red onion and tamarillo had the highest levels of phenolic compounds, while watercress and spinach extracts had the highest content of flavonoid compounds. In terms of AOA, it was found that beetroot, red onion, and tamarillo presented the highest inhibitory activity of DPPH and ABTS radicals, as well as the greatest reducing power, as demonstrated by the FRAP assays.

A quick, simple, sensitive, and reliable analytical methodology based on the QuEChERS ultrasound assisted combined with UHPLC-PDA was developed for the simultaneous determination of polyphenols in targeted fruit and vegetables. The optimized method demonstrated satisfactory results in terms of the selectivity, linearity, detection and quantification limits, precision, accuracy, and extraction efficiency. The concentrations of phenolic compounds determined in these samples showed the ability of the method to identify and quantify this type of substance in food matrices. Regarding the analyzed phenolic compounds, catechin was the most abundant polyphenol, followed by gentisic and ferulic acids, mainly in the watercress sample. In relation to flavonols, quercetin and kaempferol were found mostly in onion samples, and in small quantities in tomato and watercress. Further investigation into the isolation and identification of the responsible antioxidant components and their mechanism of action is necessary to better understand their ability to control diseases that have a significant impact on quality of life.

## Figures and Tables

**Table 1:** Total content of phenolic compounds (TPC), flavonoids (TFC), anthocyanins (TAC), and betalains (TBC) in fruits and vegetables.

Sample	TPC (mg GAE/g)	TFC (mg QE/g)	TAC (µg C3GE/g)	TBC (µg Betalains/g)
Beetroot	1.02 ± 0.01	0.97 ± 0.01	-	899.1 ± 10.9
Red onion	1.33 ± 0.09	0.47 ± 0.02	5.13 ± 0.36	-
Yellow onion	0.95 ± 0.02	0.20 ± 0.02	0.67 ± 0.24	-
Garlic	0.96 ± 0.01	0.33 ± 0.01	0.75 ± 0.12	-
Orange carrot	0.39 ± 0.02	0.26 ± 0.05	1.85 ± 0.24	-
White carrot	0.53 ± 0.01	0.76 ± 0.17	4.15 ± 0.24	-
Watercress	1.10 ± 0.03	2.55 ± 0.04	13.2 ± 3.65	-
Spinach	1.00 ± 0.02	2.52 ± 0.12	0.99 ± 0.23	-
Broccoli	0.96 ± 0.02	1.30 ± 0.03	4.10 ± 0.12	-
Tamarillo	1.10 ± 0.02	0.69 ± 0.01	17.3 ± 0.23	-
Tomato	0.52 ± 0.02	0.24 ± 0.02	1.21 ± 0.34	-

**Table 2:** Validation parameters of the QuEChERS-USAE/UHPLC-PDA methodology for the determination of polyphenols in fruits and vegetables.

No.	Analytes	Rt ^a^ (min)	λ_max_^b^ (nm)	Regression Equation	*R* ^2^	Mandel Test		LOD (µg/mL)	LOQ (µg/mL)	Concentration Level (µg/mL)	Precision (RSD%)		Trueness (Bias %)	Recovery ± σ (%)
						TV ^c^	*F* (0.95; 1; N-3) ^d^				Intra-day (*n* = 6)	Inter-day (*n* = 18)		
1	Protocatechuic acid	2.825	259	7.6389*x* − 29.253	0.9972	1.98	18.5	0.10	0.30	5	19	20	−19	65 ± 4
										10	2	14	−2	77 ± 7
										25	14	10	−11	86 ± 2
2	(+)-Catechin	3.222	278	0.9936*x* − 5.1953	0.9892	1.79	18.5	0.20	0.60	5	20	20	20	77 ± 1
										10	18	17	15	68 ± 4
										15	16	15	2	97 ± 3
3	Gentisic acid	3.856	327	0.9141*x* + 0.2776	0.9996	1.93	18.5	0.04	0.12	5	20	18	7	86 ± 11
										10	12	20	1	102 ± 7
										25	11	6	7	109 ± 2
4	Vanillic acid	4.522	261	2.2658*x* − 1.562	0.9904	1.75	18.5	0.19	0.57	5	7	8	−14	78 ± 7
										10	10	15	−14	79 ± 7
										25	13	7	−11	106 ± 2
5	Syringaldehyde	5.322	308	2.2374*x* − 2.1762	0.9981	1.94	18.5	0.08	0.25	5	16	14	−18	72 ± 13
										10	15	17	−10	88 ± 4
										25	14	8	10	104 ± 5
6	*p*-Coumaric acid	5.688	309	4.75*x* − 2.2961	0.9891	1.23	18.5	0.20	0.61	5	10	14	−20	97 ± 4
										10	9	17	−3	87 ± 2
										25	13	8	0.5	112 ± 4
7	Ferulic acid	6.038	323	2.9341*x* − 0.991	0.9925	0.91	18.5	0.17	0.50	5	4	8	2	92 ± 9
										10	14	17	−4	75 ± 21
										25	9	9	−6	103 ± 7
8	*m*-Coumaric acid	6.503	278	4.4605*x* + 5.4055	0.9930	1.87	18.5	0.16	0.48	5	11	18	−1	69 ± 8
										10	11	16	−2	86 ± 11
										25	14	7	0.3	102 ± 5
9	*o*-Coumaric acid	7.473	276	4.0666*x* + 0.831	0.9963	0.49	18.5	0.12	0.35	5	18	20	−9	59 ± 1
										10	8	9	−6	80 ± 10
										25	13	11	−4	107 ± 4
10	Cinnamic acid	9.529	277	4.4086*x* + 0.0199	0.9984	1.71	18.5	0.08	0.23	5	20	20	−20	92 ± 8
										10	11	16	−0,2	89 ± 5
										25	13	9	6	102 ± 8
11	Quercetin	9.729	372	1.75*x* + 0.6423	0.9982	1.06	18.5	0.08	0.25	5	14	19	−9	78 ± 14
										10	13	16	−4	79 ± 7
										25	14	12	10	106 ± 8
12	Kaempferol	10.484	366	2.1524x + 1.2044	0.9972	0.99	18.5	0.10	0.31	5	19	20	−5	80 ± 3
										10	10	18	−2	80 ± 3
										25	14	13	−8	105 ± 8

^a^ Rt: retention time; ^b^ **λ_max:_** maximum absorbance value obtained in PDA system detection; ^c^ TV: test value; ^d^ *F* (0.95; 1; N-3): Fisher/Snedecor F-distribution tabulated values for a 95% confidence.

**Table 3:** Concentrations of phenolic compounds found in the studied fruit and vegetable samples.

Analytes	Concentrations ± σ (µg/g Mass Fresh)										
	Beetroot	Red Onion	Yellow Onion	Garlic	Orange Carrot	White Carrot	Watercress	Spinach	Broccoli	Tamarillo	Tomato
Protocatechuic acid	n.d.	n.d.	n.d.	n.d.	4.6 ± 0.2	n.d.	7.8 ± 0.2	n.d.	n.d.	n.d.	n.d.
(+)-Catechin	n.d.	n.d.	n.d.	5.3 ± 0.01	5.3 ± 0.01	n.d.	n.d.	5.8 ± 0.03	5.6 ± 0.03	14.8 ± 0.3	7.1 ± 0.02
Gentisic acid	2.5 ± 0.2	n.d.	n.d.	n.d.	n.d.	n.d.	6.9 ± 0.2	2.7 ± 0.3	n.d.	14.8 ± 0.5	3.0 ± 0.1
Vanillic acid	n.d.	n.d.	n.d.	n.d.	1.1 ± 0.04	n.d.	n.d.	n.d.	n.d.	5.8 ± 0.1	n.d.
Seringaldehyde	1.1 ± 0.01	n.d.	n.d.	1.0 ± 0.03	1.0 ± 0.03	n.d.	n.d.	n.d.	n.d.	n.d.	n.d.
*p*-Coumaric acid	n.d.	n.d.	n.d.	n.d.	0.7 ± 0.01	n.d.	0.7 ± 0.02	n.d.	n.d.	n.d.	0.7 ± 0.01
Ferulic acid	0.5 ± 0.01	n.d.	n.d.	0.5 ± 0.04	0.6 ± 0.1	n.d.	18.0 ± 0.5	< LOQ	n.d.	n.d.	n.d.
*m*-Coumaric acid	n.d.	n.d.	n.d.	n.d.	< LOD	n.d.	n.d.	n.d.	n.d.	n.d.	< LOD
*o*-Coumaric acid	n.d.	n.d.	n.d.	n.d.	< LOD	n.d.	n.d.	< LOQ	n.d.	n.d.	n.d.
Cinnamic acid	n.d.	n.d.	n.d.	n.d.	0.4 ± 0.2	n.d.	n.d.	n.d.	n.d.	n.d.	n.d.
Quercitin	n.d.	17.1 ± 0.5	5.3 ± 0.4	n.d.	n.d.	n.d.	0.4 ± 0.03	n.d.	n.d.	n.d.	n.d.
Kaempferol	n.d.	9.2 ± 0.4	2.4 ± 0.4	n.d.	n.d.	n.d.	n.d.	n.d.	n.d.	n.d.	1.1 ± 0.03

n.d.: not detected; LOD: limit of detection; LOD: limit of quantification.

**Table 4:** Biological properties of the identified polyphenols.

Polyphenols	Chemical Structure	Bioactive Properties	Reference
Protocatechuic acid	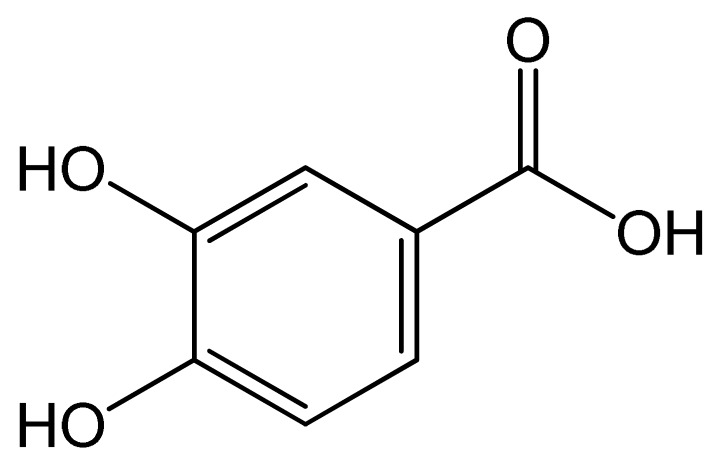	Antimicrobial, Anticancer, Antioxidant, Neuroprotective.	[69,70,71,72,73]
(+)-Catechin	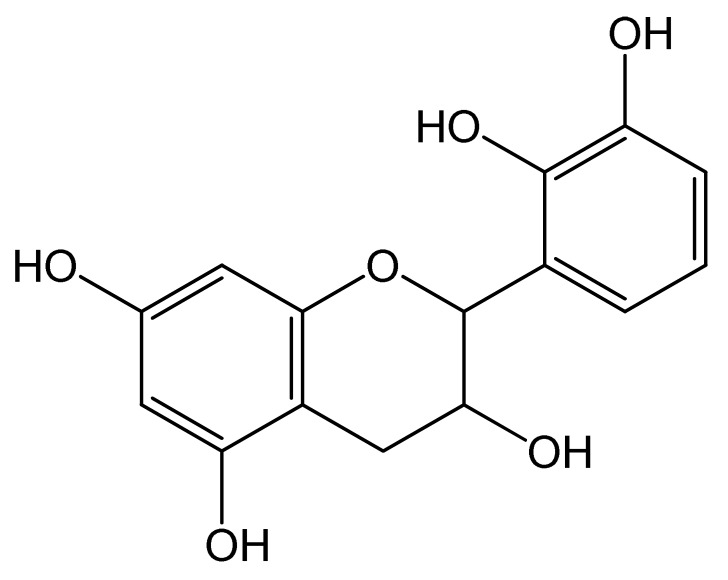	Antioxidant, Antibacterial, Anti-adipogenic, Anti-inflammatory	[74,75,76]
Gentisic acid	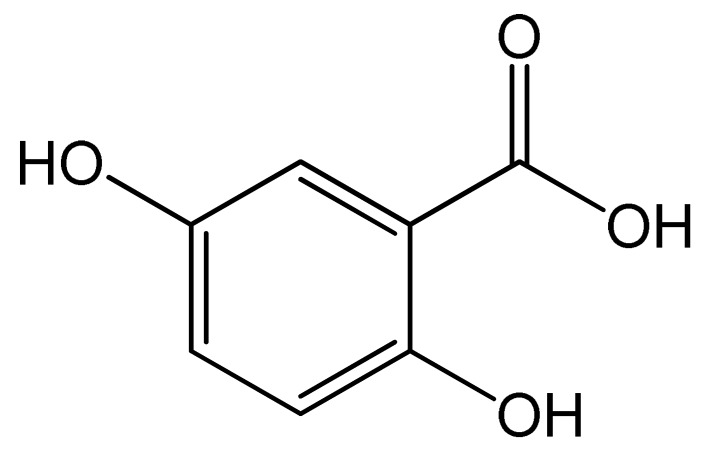	Anti-inflammatory, Antirheumatic, Analgesic activities, Antioxidant, Anticancer.	[70,77,78,79]
Vanillic acid	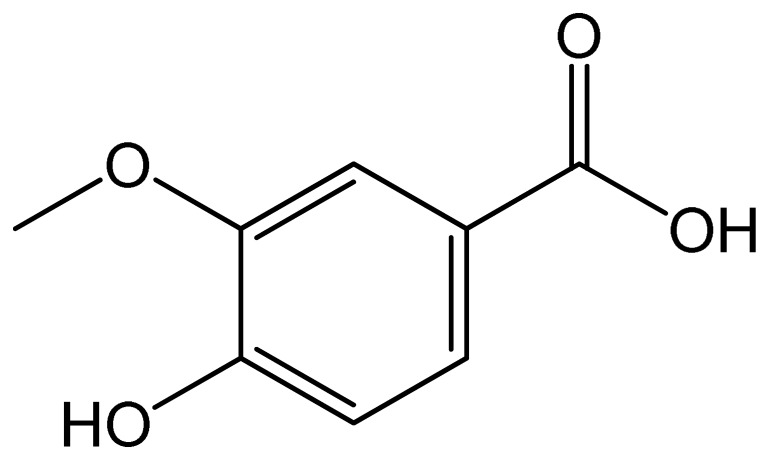	Antioxidant, Antimicrobial, Anticancer.	[80,81,82]
Syringaldehyde	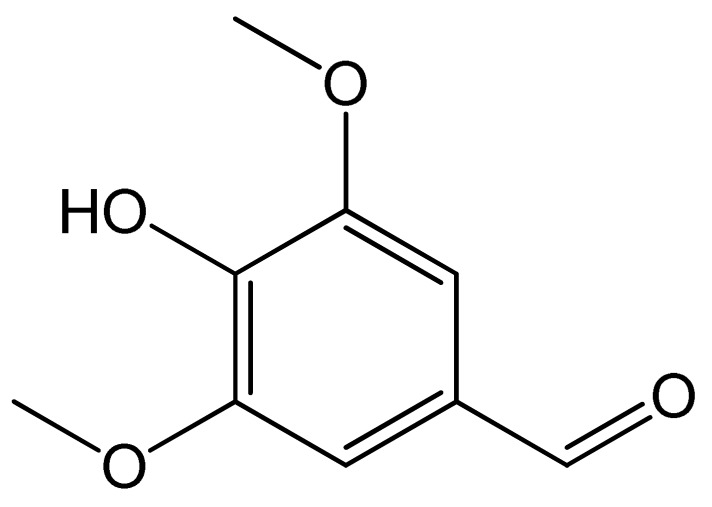	Antidiabetic, Anti-inflammatory, Antifungal, Antimicrobial	[83,84,85]
*p*-Coumaric acid	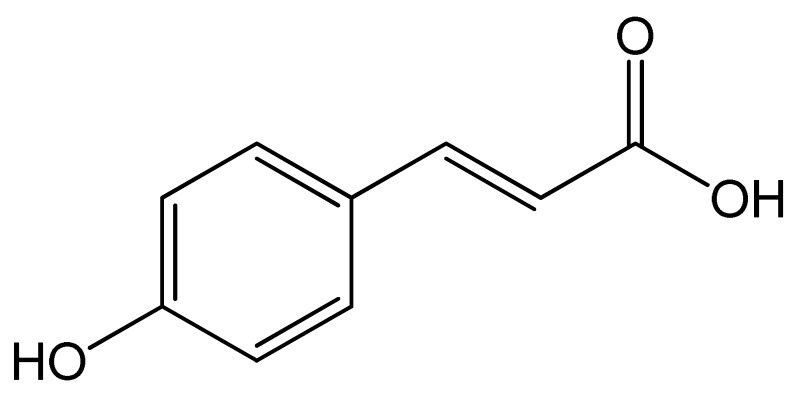	Antioxidant, Antimicrobial, Antiviral, Antimutagenic, Anticancer, Analgesic, Antipyretic, Anti-ulcer, Anti-Arthritis, Antiplatelet aggregation, Anxiolytic.	[86,87,88,89,90,91]
Ferulic acid	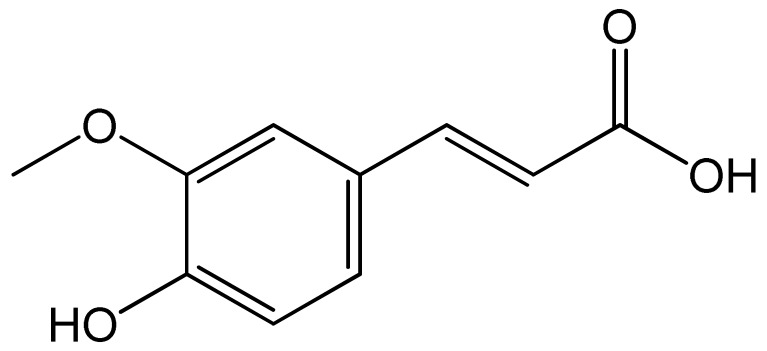	Antioxidant, Antimicrobial, Anti-inflammatory, Antidiabetic, Anticancer.	[92,93,94,95]
*m*-coumaric acid	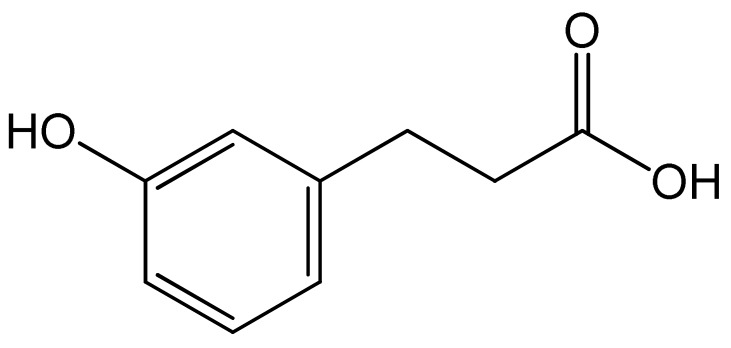	Antioxidant, Inhibitory effect on the proliferation of 3T3-L1 preadipocytes.	[96,97]
*o*-coumaric acid	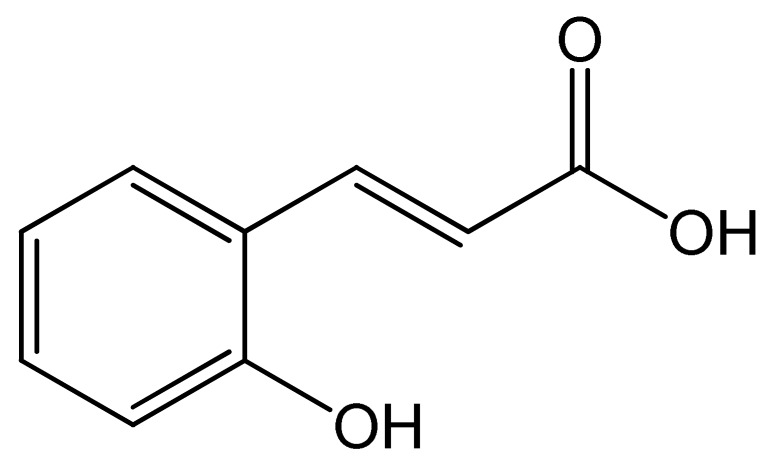	Anticarcinogenic, Antibacterial, Antialgal, Antilipidemic, Antioxidant, Anti-obesity.	[98,99,100]
Cinnamic acid	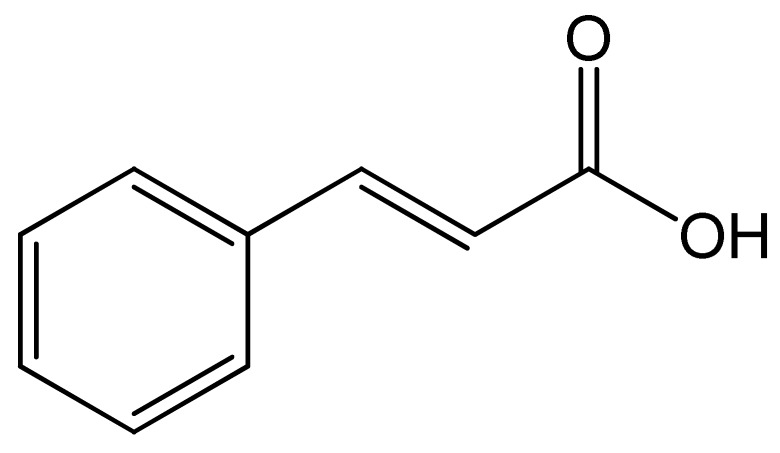	Antimicrobial, Anticancer, Antioxidant, Antibacterial	[101,102,103]
Quercetin	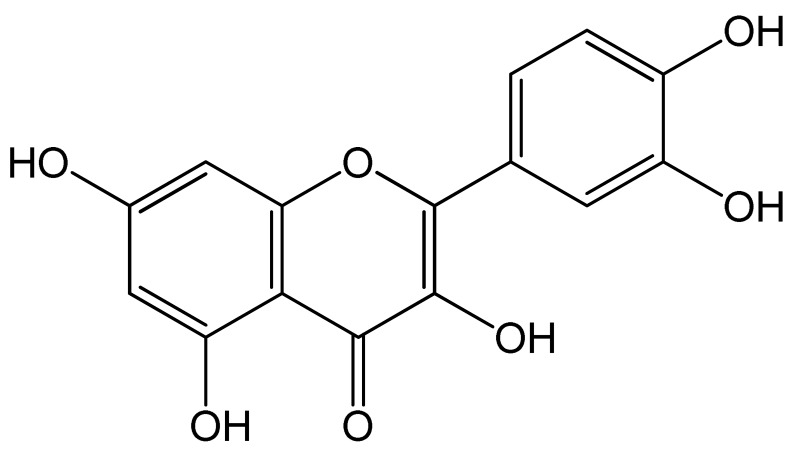	Anti-inflammatory, Anti-hypertensive, Vasodilator effect, Anti-obesity, Anti-hypercholesterolemic, Anti-atherosclerotic, Anticancer, Cytoprotective, Antidiabetic, Antioxidant, Antiviral, Antimicrobial	[104,105,106,107]
kaempferol	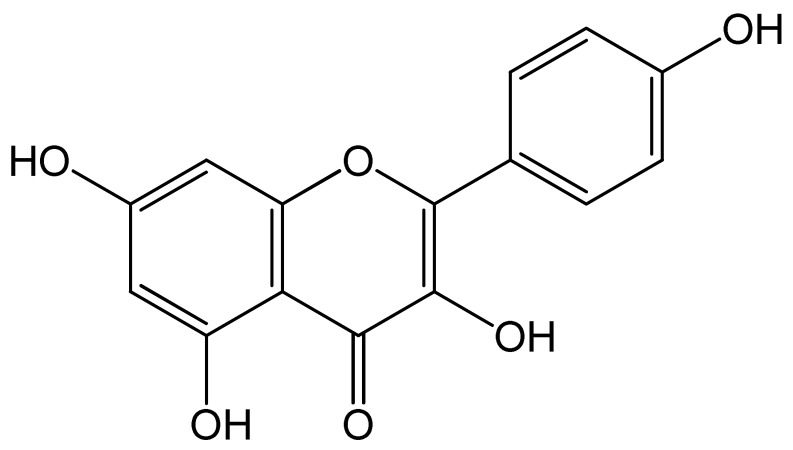	Antioxidant, Anticancer, Antiangiogenic, Neuroprotective effect, Hepatoprotective effect, Antidiabetic, Antimicrobial	[108,109,110,111]
